# Work hours as a risk factor for SARS-CoV-2 infections: cardiometabolic and sleep characteristics in rotating shift workers

**DOI:** 10.5935/1984-0063.20210013

**Published:** 2022

**Authors:** Raiza Aranha Nascimento, Virgínia Capistrano Fajardo, Luiz Antônio Alves Menezes Junior, Pedro Henrique Marques Mendonça, Maria Cristina Veiga Aranha Nascimento, Pedro Marques Oliveira Tristão, Fernando Luiz Pereira Oliveira, Raimundo Marques Nascimento Neto

**Affiliations:** 1 Belo Horizonte City Hall, Endocrinologist - Belo Horizonte - Minas Gerais - Brazil.; 2 Sciences Applied to Adult Health Care Postgraduate Program, School of Medicine, Federal University of Minas Gerais, Belo Horizonte.; 3 Postgraduate Program in Health and Nutrition, Federal of University Ouro Preto - Ouro Preto - Minas Gerais - Brazil.; 4 Postgraduate Program in Infectology and Tropical Medicine, Federal of University Minas Gerais - Belo Horizonte - Minas Gerais - Brazil.; 5 Federal of University Ouro Preto, School of Medicine - Ouro Preto - Minas Gerais - Brazil.; 6 Belo Horizonte City Hall, Cardiologist - Ouro Preto - Minas Gerais - Brazil.; 7 Federal of University Ouro Preto, Institute of Exact and Biological Sciences - Ouro Preto - Minas Gerais - Brazil.

**Keywords:** Coronavirus Infections, Betacoronavirus, Sleep Deprivation, Circadian Rhythm, Mining

## Abstract

**Objective:**

This study aims to describe the health characteristics of rotating shift mining workers that may be related to a worse course scenario for COVID-19, according to literature data.

**Methods:**

Is a cross-sectional from three studies with 1478 shift workers. Social, demographic, clinical, and biochemical variables were analyzed. Risk factors for COVID-19 analyzed: hyperglycemia, altered blood pressure, dyslipidemia, hypovitaminosis D, obesity, presence of pre-existing cardiovascular diseases, and smokers.

**Results:**

Evaluating the grouped risk factors for an unfavorable evolution of COVID-19 most workers (91.0%) presented at least one risk factor.

**Discussion:**

With coronavirus in pandemic circulation, we noticed that mineworkers are in a vulnerable position. Their exposure to occupational risk factors, to the shift system, which directly affects sleep and negatively influences immunity, added to the conditions of favorable transmissibility by the flow of people from the mines leads us to believe in their greater susceptibility to acquiring the most serious forms of the disease.

## INTRODUCTION

In late 2019, a new coronavirus (SARS-CoV-2) was identified as causing pneumonia. Subsequently, the infection spread rapidly around the world, resulting in a pandemic. So far, the only effective measure to stop the spread of the virus is social isolation, which has had major impacts on the global economy. In this context, in Brazil, mining activities were defined by the federal government as essential, that is, they maintained their activities during the COVID-19 pandemic. The Movement for Popular Sovereignty in Mining (MAM) crossed data from the National Mining Agency (ANM) with those of the health secretariat of the state of Pará and concluded that the municipalities where there are large mining projects in this state are the most affected by COVID- 19^1^.

Shift workers are subjected to changes in the circadian cycle resulting in insufficient sleep syndrome (SSI) defined as a decreased quantity or impaired quality of sleep. The American Heart Association (AHA) has recognized sleep deprivation as a cardiovascular risk factor since 2016. It is associated with central obesity, high fasting blood glucose, hypertension (HT), metabolic syndrome, sleep disorders, and immunological changes^2^. All of these factors have been well established as risk factors for the unfavorable evolution of COVID-19. As found by Peters et al. (2021)^3^ evaluating 502,493 individuals, found that increased waist circumference was associated with a higher risk of death from COVID-19 (HR: 1.36; 95% CI = 1.18-1.57). Suggesting that abdominal obesity appears to be a risk in COVID-19^3^. High glucose levels are also considered risk factors for COVID-19. Dennis et al. (2021)^4^ evaluating 19,256 patients admitted to hospital in England with COVID-19, found that patients with type 2 diabetes had a higher risk of death (HR: 1.23; 95% CI = 1.14-1.32). Another important factor to be considered is that SSI impairs immunity, making its carriers more susceptible to viral infections such as the common flu^5^.

Mining shift workers may not initially be classified as at risk for severe forms of COVID-19 because they are young and supposedly healthy individuals. However, specific characteristics of this population call the attention of the scientific community and must be carefully evaluated. Given a large number of workers with comorbidities associated with the severe form of COVID-19 and the maintenance of their activities during the pandemic, we believe it is imperative to warn about the potential risks that this represents. This data can be useful for damage containment strategies for current and future pandemics.

The study aims to describe the health characteristics of rotating shift-mining workers that may be related to a worse course scenario for COVID-19, according to literature data.

## MATERIAL AND METHODS

### Study design and population

A cross-sectional study carried out with a population of shift workers from an iron ore extraction company in two locations, in the Iron Quadrangle region, Minas Gerais, and the south of the state of Pará. The data analyzed comes from three studies carried out with workers in rotating shifts: a) the first was carried out in 2012, with 516 shift workers from four mines in the Iron Quadrangle region. A systematic sampling process was carried out to assess vitamin D (25 (OH) D), (n=391). There were losses due to incomplete data, abstaining from the answers to the questionnaire, or due to not having collected blood (n=33). Totaling 358 individuals evaluated; b) the second study was carried out in 2015 with shift workers from another mine in the Iron Quadrangle region. The population of shift workers with the position of operators was invited to participate (n=275). Of these, there were losses due to non-participation due to refusal, vacations, leave and time off (n=17); and incomplete data for not having answered the questionnaire or for not having collected blood (n=66). Totaling 192 individuals evaluated; c) the third study was carried out in 2018 on shift workers (operators and non-operators) in the southern region of Pará. Of these, there were losses due to incomplete data, for not having answered the questionnaire or for not having collected blood (n=169). Totaling 932 individuals evaluated. In all three studies, the population of shift workers with the position of operators was invited to participate. In this study, 1,482 shift workers were analyzed.

Participants of cross-sectional studies carried out in 2012, 2015 had a rotating shift schedule (4x1) of the six hours of work, followed by 12 hours of rest. After finishing the weekly four-shift cycle, they receive one day off. Participants of the cross-sectional study carried out in 2018 had a rotating shift schedule of (5x2) of the eight hours of work, followed by 24 hours of rest. After finishing the weekly five-shift cycle, they receive two days off.

### Data collect

In all studies, data collection was performed face-to-face by teams trained to administer the questionnaires, measure anthropometric data, and collect biological samples.

The social, demographic, and economic variables evaluated were: sex, age, self-reported skin color, and scholarly. Age was categorized as 20-29 years, 30-39 years, 40-49 years, 50-59 years, and 60 years or more; the self-declared skin color was categorized as White, Black, and Brown-Skinned (Black, Mulatto, Mixed or Mixed-Race), and others (Yellow or Indigenous); scholarly was categorized into up to 1^st^ degree complete, 2^nd^ degree complete, technician, graduated, and postgraduate.

The clinical evaluation was carried out employing a questionnaire about pre-existing diseases, use of medication, and smoking habits; and by measuring blood pressure. The pre-existing diseases evaluated were: hypertension (HT), diabetes mellitus (DM), dyslipidemia, cardiovascular diseases, respiratory diseases, and chronic kidney disease. The drugs evaluated were: antihypertensive, lipid-lowering, hypoglycemic agents, and antiplatelet agents. Among the antihypertensive drugs, the following classes were evaluated: angiotensin-converting enzyme (ACE) inhibitor, angiotensin receptor blocker (ARB), thiazide diuretics, beta-blockers, calcium channel blocker, and aldosterone receptor blocker. The smoking habit was categorized as a non-smoker, ex-smoker, and smoker. Blood pressure was measured in triplicate with a digital semi-automatic device. The measurement protocol followed the recommendations of the Brazilian Society of Cardiology in force in the year of data collection. Blood pressure was classified as normal (systolic blood pressure [SBP] ≤120mmHg and diastolic [DBP] ≤80mmHg), prehypertension (SBP 121-139mmHg or DBP 81-89mmHg), stage 1 hypertension (SBP 140-159mmHg or DBP 90-99mmHg), stage 2 hypertension (SBP 160-179mmHg or DBP 100-109mmHg), and stage 3 hypertension (SBP ≥180mmHg or DBP ≥110mmHg)^6^.

The evaluation of the biochemical profile was performed by analysis of the lipid profile, vitamin D, and glycemia. In the first two years, blood samples were collected after a 10-hour fast and in 2018 it was collected without a previous fast. Total cholesterol (TC), high-density lipoprotein-cholesterol (HDL), and triglycerides (TG), which were determined by the enzymatic-colorimetric method. Low-density lipoprotein-cholesterol (LDL) was calculated using Friedewald’s Equation (1972) when the concentrations of TG were <400mg/dL. The TC was classified as normal <190mg/dL, HDL >40mg/dL, TG <150mg/dL with fasting and <175 without fasting, and LDL <130mg/dL^6^. Vitamin D was determined by the chemiluminescence method and classified as sufficient 25 (OH) D >20ng/mL and hypovitaminosis D 25 (OH) D ≤20ng/mL^7^. Blood glucose was determined by fasting plasma glucose (FPG) or glycated hemoglobin (HbA1c). In the first two years, FPG was determined by the enzymatic colorimetric method, and in 2018, HbA1c was determined by the high-performance liquid chromatography method. Glycemia was classified as normoglycemia (FPG <100mg/dL or HbA1c <5.7%), risk for diabetes (FPG 100-126mg/dL or HbA1c 5.7-6.4%, and diabetes (FPG ≥126mg/dL or HbA1c ≥6.5%)^8^.

The assessment of body fat was performed by body mass index (BMI), waist circumference (WC), and neck circumference (NC). Height was measured using a stadiometer with a scale in centimeters and accuracy of one millimeter. Weight was measured on a portable body composition monitor. BMI was calculated and classified according to the World Health Organization (WHO) as underweight (BMI<18.5kg/m^2^), eutrophic (18.5-24.9kg/m^2^), overweight (25.0-29.9kg/m^2^), obese class I (30.0-34.9kg/m^2^), obese class II (35.0-39.9kg/m^2^), and obese class III (≥40.0kg/m^2^). WC was measured, in triplicate, with a simple and inelastic measuring tape at the midpoint between the iliac crest and the last costal arch, and classified as central obesity values ≥102cm in men and ≥88cm in women^9^. NC was measured at the level of the cartilage, just above the laryngeal prominence, and classified as increased values >43cm in men and >38cm in women^9^.

Participants were grouped into risk factors for COVID-19: a) altered blood glucose (pre-diabetes or diabetes) or use of antiglycemic drugs; b) altered blood pressure (stage 1 or higher hypertension) or use of antihypertensive drugs; c) dyslipidemia (TC ≥190mg/dL, or HDL ≤40mg/dL, or LDL ≥130mg/dL, or TG ≥150mg/dL) or use of lipid-lowering drugs; d) hypovitaminosis D (25 (OH) D <20ng/mL); e) obesity (BMI ≥30kg/m^2^); f) presence of pre-existing cardiovascular diseases; g) smoker (smokers and ex-smokers).

Categorical data are presented as absolute (n) and relative (%) frequencies. Continuous data presented as median and 50^th^ and 75^th^ percentiles (P25-P75), minimum and maximum. The Shapiro-Wilk test was used to verify the normality of the data. The missing data were reported in each table and were less than 5%. Descriptive analysis was performed using the Stata SE software, version 15.0 (StataCorp, College Station, TX, U.S.).

The protocol of the three studies was approved by the human research ethics committee of the Federal University of Ouro Preto (2012: CAAE No.: 0018.0.238.00-11; 2015 CAAE No.: 39682014.7.0000.5150; 2018: CAAE No.: 93760618.5.0000.5150). All participants signed an informed consent form.

## RESULTS

In a total of 1,482 shift workers mostly were men (97.4%) and black or brown (73.1%), the median age was 35.2 years (31.0-41.0), minimum of 20 years, and a maximum of 65 years, with the most prevalent age group being 30 to 39 years old (54.0%), and most of the workers (70.8%) had 2^nd^ degree complete (Table 1).

**Table 1:** Social and demographic characteristics of the 1482 workers in mining shifts.

Parameters	Classification	Total (n= 1482)
Sex	Male	1444 (97.4)
	Female	38 (2.6)
Age	20-29 years	235 (15.9)
	30-39 years	799 (54.0)
	40-49 years	342 (23.1)
	50-59 years	97 (6.5)
	≥ 60 years	8 (0.5)
Self-reported skin color	Black and brown-skinned	1081 (73.1)
	White	337 (22.8)
	Others (yellow skin and indigenous)	60 (4.1)
Scholarity	Up to 1st degree complete	58 (3.9)
	2nd degree complete	1050 (70.8)
	Technician	339 (22.9)
	Graduated	31 (2.1)
	Postgraduated	4 (0.3)

Hypovitaminosis D was observed in 18.2% (n=278). Although few declared themselves diabetic (2.9%) or were taking medication for DM (1.6%), the laboratory evaluation detected altered blood glucose in 31.3% (n=460). The same was observed with HT, in which 9.8% (n=145) reported being hypertensive and 5.9% (n=87) reported using antihypertensive medication, blood pressure measurement found that 35.9% (n=532) had some degree of HT (SBP ≥140 or DBP ≥90). Of the antihypertensive drugs used, the most reported were ARB (43.7%), ACE inhibitors (27.6%), thiazide diuretics (24.1%), and calcium channel blockers (23.0%) (Table 2).

**Table 2:** Characterization of the main risk factors for COVID-19 of the 1482 mining shift workers.

Parameters	Classification	Cutoff	Total (n= 1482)
Vitamin D	Sufficient	> 20 ng/mL	1204 (78.8)
	Hypovitaminosis D	≤ 20 ng/mL	278 (18.2)
Diabetes Mellitus	Pre-existing disease		43 (2.9)
	Use of anti-glycemic medication		23 (1.6)
Glycemia	Normoglycemia	Fasting glucose <100 mg / dL or HbA1c <5.7%	1010 (68.7)
	Risk for diabetes	Fasting glucose 100-126 mg / dL or HbA1c 5.7-6.4%	413 (28.1)
	Diabetes	Fasting glucose ≥ 126 mg / dL or HbA1c ≥ 6.5%	47 (3.2)
Arterial hypertension	Pre-existing disease		145 (9.8)
	Use of antihypertensive medication		87 (5.9)
	ACE inhibitors		24 (27.6)
	ARB		38 (43.7)
	Thiazide diuretics		21 (24.1)
	Beta-blocker		6 (6.9)
	Calcium channel blocker		20 (23.0)
	Aldosterone receptor blocker		1 (1.2)
Blood pressure	Normal	SBP ≤ 120 mmHg and DBP ≤ 80 mmHg	262 (17.7)
	Pre-hypertension	SBP 121-139 mmHg or DBP 81-89 mmHg	688 (46.4)
	Hypertension	SBP ≥ 140 mmHg or DBP ≥ 90 mmHg	532 (35.9)
	Stage 1 hypertension	SBP 140-159 mmHg or DBP 90-99 mmHg	420 (28.3)
	Stage 2 hypertension	SBP 160-179 mmHg or DBP 100-109 mmHg	90 (6.1)
	Stage 3 hypertension	SBP ≥ 180 mmHg or DBP ≥ 110 mmHg	22 (1.5)

HbA1c: glycated hemoglobin. ACE: angiotensin-converting enzyme. ARB: angiotensin receptor blocker. SBP: systolic blood pressure. DBP: diastolic blood pressure. 12 (0.81%) participants do not have data on blood glucose.

Of the anthropometric parameters, overweight (BMI ≥25kg/m^2^) was observed in 71.8% (n=1,064), and obesity in 21.1% (n=312). Waist circumference was altered in 17.4% (n=258) and neck circumference in 7.7% (n=114) (Table 3).

**Table 3:** Characterization of excess weight by anthropometric indicators of the 1482 workers in altering shifts in mining.

Parameters	Classification	Cutoff	Total (n= 1482)
Body mass index	Low weight	<18.5 kg/m^2^	7 (0.5)
	Eutrophy	18.5- 24.9 kg/m^2^	411 (27.7)
	Overweight	25.0-29.9 kg/ m^2^	752 (50.7)
	Obesity	≥ 30 kg/m^2^	312 (21.1)
	Class 1	30.0-34.9 kg/m^2^	275 (18.6)
	Class 2	35.0-39.9 kg/m^2^	34 (2.3)
	Class 3	≥ 40.0 kg/m^2^	3 (0.2)
Waist circumference	Normal	Men: ≤ 102 cm Women: ≤ 88 cm	1224 (82.6)
	Altered	Men: > 102 cm Women: > 88 cm	258 (17.4)
Neck circumference	Normal	Men: ≤ 43 cm Women: ≤ 38 cm	1368 (92.3)
	Altered	Men: > 43 cm Women: > 38 cm	114 (7.7)

Of the risk factors for COVID-19, the most prevalent were dyslipidemia (77.9%), followed by HT (37.9%), DM (31.5%), and obesity (21.1%).

When assessing the risk factors for COVID-19 groups, we observed that most shift workers (91.0%) had at least one risk factor for COVID-19. Workers with three or more risk factors for COVID-19 accounted for 32.7% (n=480) (Table 4).

**Table 4:** Risk factors for COVID-19 of the 1482 workers on mining shifts.

Parameters	Classification	Total (n= 1482)
Risk factors for COVID-19	Diabetes	463 (31,5)
	Blood pressure	561 (37.9)
	Dyslipidemia	1155 (77.9)
	Hypovitaminosis D	293 (19.8)
	Obesity	313 (21.1)
	Cardiovascular diseases	12 (0.8)
	Smoker	232 (15.7)
Risk factors for COVID-19 grouped	No risk factor	132 (9.0)
	1 risk factor	394 (26.8)
	2 risk factors	464 (31.5)
	3 risk factors	292 (19.9)
	4 risk factors	145 (9.9)
	5 risk factors	41 (2.8)
	6 risk factors	2 (0.1)

Diabetes: altered blood glucose (pre-diabetes or diabetes) or use of anti-glycemic drugs; Blood pressure: altered blood pressure (stage 1 or higher hypertension) or use of antihypertensive drugs; Dyslipidemia: TC ≥ 190 mg / dL, or HDL ≤ 40 mg / dL, or LDL ≥ 130 mg / dL, or TG ≥ 150 mg / dL or use of lipid-lowering drugs (with or without fasting); Hypovitaminosis D: 25 (OH) D < 20 ng / mL; Obesity: BMI ≥ 30 kg / m^2^; Cardiovascular diseases: pre-existing diseases; Smoker: smoker or ex-smoker.

## DISCUSSION

This study aimed to demonstrate the health characteristics of shift workers that may favor COVID-19 infection. There are no studies in the literature evaluating the occurrence of COVID-19 in these workers. This signals an important gap because we found that 91% of shift workers had at least one risk factor for COVID-19, demonstrating that they are a population at high risk for the evolution of severe forms, such as hospitalization, invasive mechanical ventilation, and/or death.

In total, 1,482 shift workers mostly were men (97.4%) and Black or Brown (73.1%) and these are some characteristics that can contribute to a worse COVID-19 infection outcome in this population. The male sex was disproportionately more affected for COVID-19 in studies in several countries^10^. In Brazil, among the cases of severe acute respiratory syndrome (SARS), 55% of Blacks and Browns died, while fewer Whites (38%). It was also observed higher education with lower lethality^11^. In the combination of race and education, inequalities were even more evident, with the highest percentage of deaths of mixed race and Black people in all levels of education. This higher mortality can be explained by the higher prevalence of HT among Blacks and/or by socioeconomic factors. In this study, shift workers are mostly men (97.4%) and Black or Brown (73.1%) and these are other characteristics that can contribute to a worse outcome in this population. Age was well established as a risk factor for severe forms and for mortality due to COVID-19^10^, which does not fit the majority of those evaluated in this study.

Most of the shift workers (71.8%) were overweight, and 21.1% (n=312) were obese. While, waist and neck circumference were altered in 17.4% (n=258), 7.7% (n=114). The organization of shift work is known to cause SSI by chronic interruption and/or reduced sleep. Consequently, there is a change in homeostasis in physiological, metabolic, and immunological functions^12^. SSI changes the levels of hormones such as leptin, ghrelin, glucagon, and melatonin^12^. Such changes generate a dysregulation in the mechanism of hunger and satiety and decrease energy expenditure leading to weight gain, glucose intolerance, and decreased insulin sensitivity. Obesity is a well-established risk factor for diabetes and cardiovascular diseases, pathologies that are associated with an increased risk for severe forms of COVID-19^10^. Besides, obesity is associated with hypercoagulation and hypoventilation, which can worsen a condition of SARS. However, in addition to these associated factors, we must remember that adipose tissue is a highly active organ that interferes with the body’s immune, endocrine, and metabolic homeostasis^13^. To date, we have no direct evidence that SARS-CoV-2 infects adipose tissue, although type 2 ACE, a receptor used by the virus to enter the cell, is present in adipose cells^13^.

Recent studies have shown that interleukin 6 (IL-6) is a strong independent predictor of mortality in patients with COVID-19. Associated with this we know that adipose tissue is the major source of IL-6 and its receptor in the human body. The hypothesis arises that adipose tissue may be a source of IL-6 activation and a trigger for the inflammatory cascade^13^. In addition to being overweight, lipid location plays an important role in the development of sleep-disordered breathing. The deposition of fat in the neck and waist is the most relevant to a greater risk of sleep-disordered breathing. There are several mechanisms, greater deposition of fat in the abdominal region predisposes to reduced diaphragm activity and the volume of inspired air. As well, due to the waist circumference reflecting the accumulation of visceral fat, much more related to sleep apnea than other forms of obesity. Also, there is a decrease in the caliber of the upper airways, caused by excess fat in the neck region. These and other factors contribute to the fact that excess adiposity favors an increased risk for respiratory sleep disorders^9^.

Hypovitaminosis D (<20ng/mL) was observed in approximately one-fifth of shift workers (19.8%). COVID-19 infection is associated with the increased production of pro-inflammatory cytokines, C-reactive protein, increased risk of pneumonia, sepsis, acute respiratory distress syndrome, and heart failure. Epidemiological and clinical studies demonstrated an inverse correlation of those characteristics with 25(OH)D concentrations^14^. Some mechanisms are proposed in the literature by which vitamin D appears to reduce the risk and severity of COVID-19: to have immune and anti-inflammatory effects, to reduce lung injury, and to reduce the risk of endothelial dysfunction. Vitamin D enhances cellular innate immunity by activating immune cells to produce antimicrobial peptides (AMPs) which lead to the destruction of envelope proteins and reduced virus replication by cathelicidin. Furthermore, vitamin D is a modulator of adaptive immunity, the calcitriol [1,25(OH)2D] suppresses responses mediated by the T helper cell type 1 (Th1) with repressing production of inflammatory cytokines; promotes cytokine production by the T helper type 2 (Th2) cells, which promotes the indirect suppression of Th1 cells; and promotes induction of the T regulatory cells, which inhibiting inflammatory processes.

The pathogenesis of COVID-19 infection involves microvascular injury induced by the inflammatory cytokine, vitamin D reduces the expression of pro-inflammatory cytokines and increases the expression of anti-inflammatory cytokines by macrophages, and also reduces the production of pro-inflammatory Th1 cytokines, such as tumor necrosis factor α (TNF-α) and interferon γ. The 1,25(OH)2D might reduce lung injury by increased surfactant production, and also prevents the constriction response of the lung blood vessel by increased expression of angiotensin-converting enzyme 2 (ACE2). Vitamin D reduces the production of reactive oxygen species and proinflammatory mediators contributing to endothelial function^14^.

A retrospective observational study of de-identified tests performed at a national clinical laboratory with 191,779 patients demonstrated an inverse relationship between circulating 25(OH)D levels and SARS-CoV-2 positivity (R-squared=0.96). Patients who had a circulating level of 25(OH)D <20ng/mL had a 54% higher positivity rate compared to those who had a blood level of 30-34ng/mL^15^. A prospective non-interventional study with 185 consecutive symptomatic SARS-CoV-2-positive patients admitted to the Medical University Hospital Heidelberg demonstrated vitamin D deficiency at admission was associated with a higher incidence of invasive mechanical ventilation and/or death (IMV/D) and worse survival. In the entire cohort (n=185), vitamin D <20ng/mL was associated with a higher risk of IMV/D (HR: 5.75; 1.73-19.09) and death (HR: 11.27; 1.48-85.55) after adjusting for age, gender, and comorbidities. In the inpatient subgroup (n=93), who had severe disease, vitamin D <20ng/mL was associated with a higher risk of IMV/D (HR: 3.99; 1.20-13.28) and death (HR: 7.97; 1.05-60.60) after adjusting^16^. Shift workers, due to erratic sun exposure, are at high risk for vitamin D insufficiency, which might favor COVID-19 infection, which is potentially reversible with adequate monitoring and supplementation.

Of the risk factors for COVID-19 considered in this study, dyslipidemia was the most prevalent, affecting nearly four-fifths of shift workers (77.9%). A meta-analysis by Atmosudigdo et al. (2021)^17^ evaluating 9 studies with 3663 patients, found that subgroup analysis showed that dyslipidemia was associated with severe COVID-19 (RR: 1.39; 95% IC = 1.03-1.87; I2: 57.4%, *p*=.029). And the association was stronger in older patients, male, and hypertensive^17^. This poor outcome for COVID-19 mortality may be explained by the accumulation of cholesterol in macrophages and other immune system cells in individuals with dyslipidemia, which stimulates inflammatory responses. Or even endothelial dysfunction, common in dyslipidemia, which predisposes the patient to CVD-related complications that increase mortality in COVID-19^17^.

In the laboratory evaluation 28.1% (n=413) had pre-diabetes criteria and 3.2% (n=47) were diagnosed as diabetic. A Brazilian study elucidated the possible explanations for the greater severity of COVID-19 in diabetics^18^. Elevated glucose levels are known to directly induce viral replication and the expression of pro-inflammatory cytokines. The authors demonstrated that infected monocytes promote the dysfunction of T-cells and the death of pulmonary epithelial cells. These data may explain why decompensated diabetes can lead to impaired adaptive immune response and pulmonary dysfunction in patients with severe COVID-19.

Hypertension appears to be the comorbidity most often associated with severe cases of COVID-19^10^. In the current study, 9.8% (n=145) of the workers declared themselves to be hypertensive and 5.9% (n=87) claimed to use antihypertensive medication. After blood pressure measurement, 35.9% (n=532) showed some degree of hypertension (SBP ≥140 or DBP ≥90). Brazilian epidemiological data indicate that 14.5% and 24.8% of men in Pará and Minas Gerais, respectively, have HT^19^. Thus, we observed an alarmingly higher prevalence of HT among shift workers when compared to the population of the same age and gender. Also, of the antihypertensive drugs used, the most reported were ARB (43.7%), ACE inhibitors (27.6%), thiazide diuretics (24.1%), and calcium channel blockers (23.0%). However, there is no evidence to support an association between the use of antihypertensives such as renin-angiotensin system inhibitors or ARB, and the severity of COVID-19^20^. Suggesting that the high mortality of COVID-19 in hypertensive patients is due to the cardiovascular disease itself and not to its treatment^20^.

Evaluating the risk factors grouped for an unfavorable evolution of COVID-19 (altered glycemia, altered blood pressure, dyslipidemia, hypovitaminosis D, obesity, presence of pre-existing cardiovascular diseases, and smoking), we observed that only 9.0% (n=132) of the workers evaluated did not present any risk factor. Workers with three or more risk factors for COVID-19 represented 32.7% (n=480) (Table 4). Alternating shift workers have a high prevalence of SSI, which in addition to being a risk factor for DM and obesity, alters immunological markers such as lymphocytes and natural killer cells and increases inflammatory cytokines such as interleukin 6 (IL-6) and tumor necrosis factor α (TNF-α)^5^. Severe cases of COVID-19 are attributed to an exuberant inflammatory response, with persistent fever, inflammatory markers, and elevated proinflammatory cytokines. These features frame COVID-19 as a cytokine storm syndrome. This syndrome is characterized by an unregulated immune response that leads to multiple organ failure. People with SSI already have a chronically activated immune system and may be at high risk of developing this severe form of COVID-19.

Limitations of this study include do not have information related to the occurrence of COVID-19 among shift workers. However, research on risk factors for COVID-19 in this population is still limited. Second, a cross-sectional design is not the best design for establishing the risks of COVID-19 associated with shift work exposure, but without some preliminary identification of transversal associations, longitudinal studies assessing an increased risk for COVID-19 in shift workers are unlikely to be conducted.

The strengths of this study include a large sample size of shift workers. And with extremely important results that can help in the implementation of adequate preventive approaches to reduce the impact of these diseases and possible complications of COVID-19 in this group of workers.

Literature data indicate that shift workers are at higher risk for common infections with colds and gastroenteritis^5^. It is believed that sleep can influence the immune system via the hypothalamus-pituitary-adrenal axis (HHA) and through the sympathetic nervous system. A night of sleep deprivation activates the HHA axis, raising plasma cortisol. The latter reduces the expression of several genes that encode pro-inflammatory cytokines. It is also known that glial neurons and immune cells share common intercellular signals^5^. Based on these data, the hypothesis that shift workers are more susceptible to infection by SARS-CoV-2 is plausible and should be taken into account in public health measures.

In addition to the biological factors, which were the subject of this study, it is also necessary to evaluate the occupational factors in which these individuals are inserted. This is because individuals with occupations that require close interpersonal interaction are considered to be at higher risk of acquiring COVID-19. Especially if they are considered essential, such as mining, where there is no possibility of maintaining total social isolation.

In the state of Pará, where part of the study was conducted, data from the health secretariat showed that municipalities, where there are large mining projects in this state, are the most affected by COVID-19^1^. At the beginning of the pandemic, until May 20^th^, 610 contaminations and 49 deaths had been confirmed (lethality rate of 8.0%); in Canaã dos Carajás, already in Marabá, the numbers are even more frightening because of the high percentage of lethality, since 280 contaminations with 53 deaths have been identified, that is, an astounding lethality rate of 18.9%, much higher than the national average.

A review study conducted by the European Centre for Disease Prevention (ECDC-19), examining whether outbreaks in occupational settings in the European economic area and the United Kingdom were associated with occupational clusters, reported that factors potentially related to a higher risk of infection by SARS-CoV-2 may be the contact of workers in closed environments, such as in the use of elevators and shared bathrooms^21^. Besides, shift workers in the mining industry are frequently submitted to agglomerations in the work routine, such as in lodgings, shift changes, and in the transportation provided by the company, which can vary from 5 to 46 passengers in cars and buses, respectively, in trips that can last up to 2 hours. In addition to the cafeterias, the self-service model presents a great risk of food contamination with droplets of saliva and the use of common serving utensils and should be abandoned. This can be a great source of contamination.

During a pandemic, managers should provide good transportation conditions that respect the guidance of social distancing, in addition to the constant sanitation of places with a high turnover of people. In the cafeterias, the self-service model should be abandoned due to the high risk of food contamination with partridges and the use of common serving utensils.

In this context, mining shift workers may not initially be classified as at risk for severe forms of COVID-19 because they are young and supposedly healthy individuals. Although their exposure to occupational risk factors, to the shift system, which directly affects sleep and negatively influences immunity, added to the individual’s health conditions of favorable infection to COVID-19 leads us to believe in their greater susceptibility to acquiring the most serious forms of the disease. The specific characteristics of this population call the attention of the scientific community and must be carefully evaluated in future studies.
